# The relationship between commercial cotton cultivars with varying *Meloidogyne incognita* resistance genes and yield

**DOI:** 10.21307/jofnem-2020-064

**Published:** 2020-07-29

**Authors:** Terry A. Wheeler, Kerry Siders, Cecilia Monclova-Santana, Jane K. Dever

**Affiliations:** 1 Texas A&M AgriLife Research, Lubbock, TX; 2 Texas A&M AgriLife Extension Service, Levelland, TX; 3 Texas A&M AgriLife Extension Service, Lubbock, TX

**Keywords:** Cotton, *Gossypium hirsutum*, *Meloidogyne incognita*, Resistance, Southern root-knot nematode

## Abstract

Small plot cotton cultivar trials (12 trials) were conducted from 2016 to 2019 in fields infested with *Meloidogyne incognita*. Entries in these trials included commercial cultivars with partial and high resistance to *M*. *incognita*, as well as cultivars with no known resistance. Different resistant groups were created based on different cotton seed companies and their descriptions of the *M*. *incognita* resistant cultivars. Groups were none (susceptible); partial resistance found in Stoneville or Fibermax cultivars (PR-FM/ST); partial resistance found in PhytoGen cultivars (PR-PHY); resistance (unknown gene(s)) in Deltapine cultivars (NR-DP); and highly resistant cultivars homozygous for *RK1* and *RK2* resistant genes in PhytoGen cultivars (HR-PHY). The highest lint yields using a mixed model analysis were found in the PR-FM/ST (1,396 kg lint/ha), HR-PHY (1,327 kg lint/ha), and PR-PHY (1,314 kg lint/ha) groups. Yield for NR-DP (1,234 kg lint/ha) was not different (*p* > 0.05) than yield for susceptible cultivars (1,243 kg lint/ha). If the older resistant cultivars from Deltapine and PhytoGen (those with only Roundup Ready® herbicide technology) were removed from the analysis, then HR-PHY yields increased by 133 kg of lint/ha to 1,460 kg lint/ha and NR-DP yields remained approximately unchanged (1,227 kg lint/ha). Newer HR-PHY had much improved yield over the first HR-PHY cultivars. Newer HR-PHY averaged 17% higher yield than the susceptible group. LOG_10_ (*M*. *incognita* eggs/500 cm^3^ soil + 1) were highest for the susceptible cultivars (3.2), followed by PR-FM/ST (2.6), NR-DP (2.4), PR-PHY (2.1), and lowest with HR-PHY (1.4). The newer HR-PHY cultivars (those with ENLIST® herbicide technology) combine excellent yields (17% higher than susceptible cultivars) with high (96%) suppression of *M*. *incognita*.

The southern root-knot nematode, *Meloidogyne incognita* (Kofoid and White) Chitwood, is widely distributed across the southern USA. In the Southern High Plains of Texas, *M*. *incognita* infested 40 to 50% of the cotton fields (Starr et al., 1993; Wheeler et al., 2000). In the absence of nematode management tactics, it is estimated that *M*. *incognita* reduces yield in the west Texas area by an average of 26% (Orr and Robinson, 1984). Management options for this nematode include crop rotation, nematicides, and host resistance. Crop rotation options are limited since *M*. *incognita* has a wide host range. The granular nematicide aldicarb was at one time heavily utilized by producers in the Southern High Plains (Wheeler et al., 2000), but production ceased in 2011 and producers shifted to other chemical options. Other chemical nematicides are currently available on cotton as seed treatments (abamectin, thiodicarb, and fluopyram) or liquid in-furrow (fluopyram), at-plant applications. However, the water solubility of these products is much lower than aldicarb (Faske and Brown, 2019) and poorly suited for a drier environment where most of the nematicide may be left on the seed coat (Faske and Starr, 2007). There has been an increase in the number of cotton cultivars with *M*. *incognita* resistance or tolerance in recent years (Wheeler et al., 2018).

The development of *M*. *incognita* resistance in many commercial cotton cultivars can be initially traced to Auburn 623 RNR (Shepherd, 1974). It has been demonstrated that there are two genes associated with the root-knot nematode resistance in Auburn 623 RNR (Gutiérrez et al., 2010). One gene which is located on chromosome 11 is associated with a reduction in the number of galls and it is involved with a delay or reduction in sedentary second-stage juveniles (J2) which develop in later life stages (Da Silva et al., 2019; Wubben et al., 2020). The second gene, which is located on the small arm of chromosome 14, is associated with a reduction in *M*. *incognita* reproduction rate, by reducing the total number of females and compromised egg production in females (Da Silva et al., 2019; Wubben et al., 2020). The combination of both genes resulted in a synergism with regard to nematode suppression.

Seed companies that offer cotton cultivars advertised with *M*. *incognita* resistance or tolerance include BASF with the Fibermax® and Stoneville® brands, Bayer CropSciencs with the Deltapine® brand, and Corteva™ Agriscience with the PhytoGen® brand. The objective of this study was to compare differences in *M*. *incognita* density and cotton lint yield with susceptible and partially or highly *M*. *incognita* resistant cultivars from different companies.

## Materials and methods

### Field details

Trials were conducted in 12 site years, naturally infested with *M*. *incognita* (race 4) (Wheeler et al., 2018) from 2016 to 2019. Due to the intense monocropping with cotton, none of the fields had mixed species of *Meloidogyne*. Test sites were in Dawson, Gaines, and Terry counties in 2016; Dawson, Gaines, Hockley, and Terry counties in 2017; Dawson and Hockley counties in 2018; and Dawson, Hall, and Lynn counties in 2019. Plots were two-row wide, 11  m long, and on 1  m centers. Each trial had entries arranged in a randomized complete block design with four to six replications. All trials were on irrigated (deficit irrigated) fields. Tests were planted using a cone planter, and seed was packaged with 144 seed/plot row (13 seed/meter row). The plots were mechanically harvested with a cotton stripper designed to weigh the plot yield on load cells. A 1,000 g sample was collected from harvested plots and two replications were ginned from each entry to determine lint percentage of the harvested cotton.

### Nematode sampling

Plots were soil sampled in August or early September to assay for root-knot nematode. Samples consisted of 5 cores/plot collected with a narrow-bladed (40 cm depth, 15 cm width at top, and 8 cm width at the bottom) shovel to a depth of 20 cm, close to the taproot. The top 6 cm of soil was discarded and then soil from 6 to 20 cm depth, including some roots, was removed. The soil was mixed in a bucket and then a subsample of 1,000 cm^3^ soil was removed and placed in a plastic bag. The soil samples were refrigerated for < 2 weeks before being assayed for root-knot nematodes. A pie-pan assay with 200 cm^3^ soil + root fragments was used to extract second-stage juveniles (J2) over 48 h (Thistlethwayte, 1970). The circular pie-pans are made of glass with 18 cm diameter at the base, 22 cm at the top, and 3 cm tall. Three washers were placed in the base of the pie-pan and wire mesh (0.64 cm diameter) laid on the top. Two pieces of Kleenex (2-ply) were laid on top of the mesh and then the soil sample was placed on the Kleenex. Tap water (250 ml) was gently added to the pie-pan without disturbing the soil, and then the wet Kleenex was arranged around the soil to hold it out of the water. A Styrofoam cover was placed over the pie-pan to eliminate evaporation. The extracted J2 were enumerated by concentrating the extracted liquid to 100 ml and then counting a 5 ml aliquot. This assay is only effective on mobile and live nematodes. A second assay with 500 cm^3^ soil was used to extract root-knot nematode eggs. The soil + root fragments were placed in a bucket with 3 L of water and stirred for 10 s. After allowing to settle for 15 s, the contents were poured over a sieve with a pore size of 230 µm and the root fragments caught on the sieve were washed into a beaker in 100 ml tap water and mixed on a stir plate for 5 min in NaOCl (0.525%) (Hussey and Barker, 1973). The mixture was poured through a sieve with a pore size of 230 µm stacked over a sieve with a pore size of 25 µm. The contents from the bottom sieve were rinsed with tap water, washed into a beaker (150 ml of water), and the eggs were enumerated from a 5 ml aliquot. The first sieving step with a pore size of 230 µm, which catches only larger sized material like root fragments, would eliminate all the singly laid nematode eggs, typical of vermiform-shaped nematodes. In the second step, the NaOCl extraction breaks down the *Meloidogyne* egg sacks attached to the roots, and then the sieve with a 25 µm pore size catches many of the eggs that were released from the egg sacks.

### Cotton cultivars

*Meloidogyne incognita* resistant cultivars were placed in the following groups: (i) FM/ST partial resistance (PR-FM/ST): ‘ST 4946GLB2’, ‘FM 2011GT’, ‘FM 1911GLT’, and ‘FM 1621GL’; (ii) Deltapine (NR-DP): ‘DP 1454NR B2RF’, ‘DP 1558NR B2RF’, ‘DP 1747NR B2XF’, and ‘DP 1823NR B2XF’; (iii) PhytoGen partial (PR-PHY): ‘PHY 250 W3FE’, ‘PHY 320 W3FE’, ‘PHY 350 W3FE’, ‘PHY 400 W3FE’, ‘PHY 430 W3FE’, and ‘PHY 440W3FE’; and (iii) PhytoGen highly resistant (HR-PHY): ‘PHY 417 WRF’, ‘PHY 480 W3FE’, ‘PHY 500 W3FE’, and ‘PHY 580 W3FE’ (Table 1). The number of tests and years a cultivar was tested are presented in Table 1.

**Table 1.** Cultivars^1^ grouped into *Meloidogyne incognita* (*Mi*) resistance categories^2^ for analysis.

Cultivar	*Mi* category^b^	Trials	Years
DP 1454NR B2RF	NR	2	2016
DP 1558NR B2RF	NR	9	2016 to 2017
DP 1747NR B2XF	NR	11	2016 to 2019
DP 1823NR B2XF	NR	3	2018 to 2019
FM 1621GL	PR	3	2018 to 2019
FM 1911GLT	PR	12	2016 to 2019
FM 2011GT	PR	8	2016 to 2018
PHY 250 W3FE	PR	1	2019
PHY 320 W3FE	PR	4	2018 to 2019
PHY 350 W3FE	PR	2	2018
PHY 400 W3FE	PR	4	2018 to 2019
PHY 417 WRF	HR	8	2016, 2017
PHY 430 W3FE	PR	1	2018
PHY 440 W3FE	PR	3	2018
PHY 480 W3FE	HR	6	2017 to 2019
PHY 500 W3FE	HR	2	2019
PHY 580 W3FE	HR	2	2019
ST 4946GLB2	PR	13	2016 to 2019

**Notes:** ^1^Susceptible cultivars included in these trials are listed and the number of trials in (): Croplan Genetics (CP) ‘CP 3475 B2XF’ (1), ‘CP 3885 B2XF’ (1), ‘CP 9178 B3XF’ (2); ‘CP 9598 B3XF’ (1), ‘CP 9608 B3XF’ (1); Deltapine (DP) ‘DP 1522 B2XF’ (6), ‘DP 1612 B2XF’ (1), ‘DP 1646 B2XF’ (3), ‘DP 1820 B3XF’ (3), ‘DP 1822 XF’ (3), ‘DP 1840 B3XF’ (2), ‘DP 1845 B3XF’ (1), ‘DP 1851 B3XF’ (1), ‘DP 1908 B3XF’ (1), ‘DP 1909 XF’ (2), ‘DP 1916 B3XF’ (1), ‘DP 1948 B3XF’ (1); Fibermax (FM) ‘FM 1320 GL’ (1), ‘FM 1888 GL’ (2), ‘FM 1953 GLTP’ (1), ‘FM 2322 GL’ (1), ‘FM 2398 GLTP’ (4), ‘FM 2498 GLT’ (4), ‘FM 2574 GLT’ (4); NexGen (NG) ‘NG 2982 B3XF’ (1), ‘NG 3406 B2XF’ (7), ‘NG 3500 XF’ (4), ‘NG 3640 XF’ (2), ‘NG 3699 B3XF’ (1), ‘NG 3930 B3XF’ (1), ‘NG 3956 B3XF’ (1), ‘NG 3994 B3XF’ (1), ‘NG 4545 B2XF’ (3), ‘NG 4689 B2XF’ (4), ‘NG 4777 B2XF’ (2), ‘NG 4936 B3XF’ (2); PhytoGen (PHY) ‘PHY 210 W3FE’ (1), ‘PHY 300 W3FE’ (1), ‘PHY 330 W3FE’ (1), ‘PHY 333 WRF’ (1), ‘PHY 340 W3FE’ (1), ‘PHY 450 W3FE (1)’, ‘PHY 490 W3FE (2)’, ‘PHY 499 WRF’ (7); and Stoneville (ST) ‘ST 4550 GLTP’ (3), ‘ST 5020 GLT’ (1), ‘ST 5122 GLT’ (1), ‘ST 5471 GLTP’ (1), ‘ST 5707 B2XF’ (2). ^2^HR-PHY are PhytoGen cultivars that have two-gene homozygous *M*. *incognita* resistance; PR-PHY are PhytoGen cultivars with partial resistance to *M*. *incognita*; NR-DP are Deltapine cultivars with *M*. *incognita* resistance; PR-FM/ST are Fibermax and Stoneville cultivars with partial resistance to *M*. *incognita*.

The cultivar ST 4946GLB2 (PVP #201300350) was listed as moderately resistant to *M*. *incognita* in its plant variety protection (PVP) certificate. FM 2011GT (PVP #201100382) did not list any testing with *M*. *incognita* on its PVP certificate; however, in the BASF Cotton Variety Catalog (2020), it is listed as tolerant to root-knot nematode. FM 1911GLT (PVP #201600319) was developed with FM 2011GT as the recurrent parent in a backcross program. It is listed as moderately resistant to *M*. *incognita* on its PVP certificate, or tolerant to root-knot nematode in its variety guide (BASF Cotton Variety Catalog, 2020). FM 1621GL which was first available commercially in 2019 is listed as root-knot nematode tolerant (BASF Cotton Variety Catalog, 2020). The cultivar ST 5600  B2XF which was first available commercially in 2019 is listed as root-knot nematode resistant (BASF Cotton Variety Catalog, 2020). Given the stronger resistant designation as opposed to tolerant for other BASF cultivars, it is likely that ST 5600 B2XF requires a separate group than the other BASF cultivars. Since it was only planted in one trial, it was eliminated from the analysis.

The cultivar DP 1454NR B2RF (PVP #201400054) contained the RKN1 and RKN2 genes according to its PVP certificate and was developed by a marker assisted backcross program using their elite RKN resistant line ‘10Y0402’. DP 1558NR B2RF (PVP #201400513) and DP 1747NR B2XF (PVP #201700046) also list 10Y0402 as the recurrent parent in a backcross program. However, on their PVP certificates, they were only genotyped for the RKN1 gene. DP 1823NR B2XF was first available commercially in 2018 and was described as resistant to root-knot nematode (Albers and Gholston, 2018).

The first PhytoGen cultivars with high resistance to *M*. *incognita* were PHY 417 WRF and PHY 427 WRF (Fuchs et al., 2015). PHY 480 W3FE, PHY 500 W3FE, and PHY 580 W3FE are homozygous for the *RK1* and *RK2* genes (Lege, personal communication). PHY 250 W3FE, PHY 320 W3FE, PHY 350 W3FE, PHY 440 W3FE, PHY 430 W3FE, and PHY 440 W3FE do have at least one RK gene in either a homozygous or heterozygous state, but do not have the *RK1* and *RK2* genes in a homozygous state (K. Lege, personal communication). They are therefore placed in the PR-PHY category.

### Analysis

A mixed model analysis (SAS version 9.4, SAS Institute, Cary, NC) was conducted with lint yield, LOG_10_ (*M*. *incognita* eggs/500 cm^3^ soil + 1) (LEggs) and J2 of *M*. *incognita*/200 cm^3^ soil. The model random terms were: year, site(year), block(year site), year × group, site × group(year), and block × group(year site). An analysis was conducted on lint yield, LEggs, and J2 as the dependent variables and group as the independent variable. A second mixed model analysis was conducted on the three dependent variables which omitted the cultivar PHY 417 WRF from the HR-PHY group and DP 1454NR B2RF and DP 1558NR B2RF from the NR-DP group. The least square means for the different resistance groups were compared with pairwise tests using the *t*-test at *p* < 0.05.

## Results

Lint yield, for the analysis of the entire data set, was higher for PR-FM/ST (1,396 kg lint/ha) than for susceptible (1,243 kg lint/ha) and NR-DP (1,234 kg lint/ha) groups (Table 2). When PHY 417 WRF, DP 1454NR B2RF, and DP 1558NR B2RF were removed from the analysis, then lint yield was higher for HR-PHY (1,460 kg lint/ha) and PR-FM/ST than for susceptible and NR-DP (1,227 kg lint/ha) groups (Table 2). PR-PHY had intermediate yields. The newer HR-PHY cultivars all contain the ENLIST® herbicide technology and the newer NR-DP cultivars contain the dicamba-tolerant herbicide technology. HR-PHY (with ENLIST traits) yielded 17% more than susceptible cotton cultivars.

**Table 2.** Least square means^1^ of nematode resistance groups for lint yield, *Meloidogyne incognita* eggs and second-stage juveniles (J2).

	Analysis for full data set				Omitting data for older cultivars^4^			
R group^2^	Lint yield (kg/ha)	Eggs/500 cm^3^ soil	LE3	J2/200 cm^3^ soil	Lint yield (kg/ha)	Eggs/500 cm^3^ soil	LE	J2/200 cm^3^ soil
HR-PHY	1,327 ab^2^	154	1.5 d	27 b	1,460 a	340	1.4 d	27 b
PR-PHY	1,314 ab	2,071	2.1 c	59 b	1,330 ab	2,193	2.1 c	32 b
NR-DP	1,234 b	1,456	2.4 bc	76 b	1,227 b	1,880	2.4 bc	37 b
PR-FM/ST	1,396 a	3,310	2.7 b	146 b	1,396 a	3,283	2.6 b	73 b
None	1,243 b	7,871	3.3 a	336 a	1,244 b	7,867	3.2 a	168 a

**Notes:** ^1^Least square means followed by the same letters are not significantly different at *p* = 0.05, based on a mixed model analysis and *t*-test (pairwise mean) comparisons. ^2^HR-PHY are PhytoGen cultivars that have two-gene homozygous *M*. *incognita* resistance; PR-PHY are PhytoGen cultivars with partial resistance to *M*. *incognita*; NR-DP are Deltapine cultivars with *M*. *incognita* resistance; PR-FM/ST are Fibermax and Stoneville cultivars with partial resistance to *M*. *incognita*. ^3^LE = LOG_10_(*M*. *incognita* eggs/500 cm^3^ soil +1). ^4^The cultivars DP 1454NRB2RF, DP 1558NRB2RF, and PHY 417WRF were omitted from the analyses.

HR-PHY cultivars were more resistant to *M*. *incognita* than all other resistance categories, based on the LOG_10_ transformed egg density (LEgg, Table 2). LEgg was 1.49 for HR-PHY cultivars, 2.12 for PR-PHY cultivars, 2.41 for NR-DP cultivars, 2.65 for PR-FM/ST cultivars, and 3.26 for susceptible cultivars (Table 2). Removing PHY 417 WRF, DP 1454NR B2RF, and DP 1558NR B2RF from the analysis did not change the LEggs substantially. J2 were higher for susceptible cultivars than for all cultivars with *M*. *incognita* resistance (Table 2). Most of the soil population density of *M*. *incognita* was associated with eggs in the root fragments rather than J2 in the soil.

## Discussion

Management of root-knot nematode in cotton with resistant cultivars is an inexpensive and safe option for producers. There are many commercial cotton cultivars available with at least partial resistance to *M*. *incognita*, though the percent of hectares planted with these resistant cultivars (Agricultural Marketing Service – Cotton and Tobacco Program, 2019) remains well below the infested area for cotton (National Cotton Council, 2020). The results presented here clearly show the benefits in terms of yield and reduction of *M*. *incognita* density of currently available cultivars with at least partial resistance to *M*. *incognita*. The HR-PHY resistance, which involves two well described resistance genes (*RK1* and *RK2*) (Da Silva et al., 2019; Gutiérrez et al., 2010; Wubben et al., 2020), in commercial cultivars was superior to commercial cultivars with partial resistance or the DeltaPine source(s) of resistance.

The first developed germplasm with high levels of *M*. *incognita* resistance in agronomically advanced cotton was Auburn 623 RNR (Shepherd, 1974). The first commercial cultivar developed with partial *M*. *incognita* resistance was LA887 (Jones et al., 1991), which was marketed under the Stoneville brand. The earliest transgenic *M*. *incognita* partially resistant cultivar, ST 5599BR (PVP #200300279) was created by a backcross program where LA887 was the recurrent parent. ST 5599BR was planted on <2% of the cotton in Texas (Agricultural Marketing Service – Cotton and Tobacco Program, 2003, 2004, 2005, 2006, 2007, 2008). In the Southern High Plains of Texas, approximately 40 to 50% of the cotton acreage (1.4 million ha) is infested with *M*. *incognita* (Starr et al., 1993; Wheeler et al., 2000).

One of the first PhytoGen cultivars developed with high resistance to *M*. *incognita* was PHY 417 WRF (Fuchs et al., 2015). This cultivar had relatively poor yield potential. More recently developed cultivars from PhytoGen with *M*. *incognita* resistance and the ENLIST™ herbicide trait had much better yield potential. This was evident in the increase in average lint yield that occurred for the HR-PHY group when PHY 417 WRF was removed from the analysis. The resistance to *M*. *incognita* in all HR-PHY cultivars was excellent.

The NR-DP cultivars did not have higher yields in the Southern High Plains of Texas than *M*. *incognita* susceptible cultivars, though they did reduce *M*. *incognita* egg density by 82%. All the NR-DP cultivars, except DP 1823NR B2XF, require a long growing season to maximize their yield potential. The Southern High Plains of Texas typically do not have enough heat units (HU) to mature out long-season cotton cultivars, and instead favors early medium to medium maturity cultivars. The HR-PHY group also included several long-season cotton cultivars (PHY 500 W3FE and PHY 580 W3FE), but these were only tested in 2019. The combined cotton HU (DD60) from 27 May to 30 September in Dawson county, TX (which are representative for most trial locations) in 2019 were 2,585. This was much higher than combined HU over the same dates and location in 2016 (2,285 HU), 2017 (2,277 HU), and 2018 (2,495 HU) (West Texas Mesonet, 2016-2019).

While the type and consistency of *M*. *incognita* resistance genes did not appear to affect tolerance in resistant cultivars (partial resistant versus highly resistant cultivars could yield well), they did affect *M*. *incognita* density. The synergism that is expected when both the *M*. *incognita* resistant genes are present (Da Silva et al., 2019) would describe the low *M*. *incognita* densities found with HR-PHY group. The next level of *M*. *incognita* resistance was found with PR-PHY and NR-DP. The PR-PHY group did not have both *RK1* and *RK2* genes, described by Gutiérrez et al. (2010) as homozygous in the populations. However, they did contain one or both these genes in at least a heterozygous state in the population. The plant variety protection certificate for DP 1454NR B2RF stated that *RK1* and *RK2* were homozygous in the population. It is not known if both *RK1* and *RK2* genes were the same referenced by Gutiérrez et al. (2010). Only *RK1* gene was described as homozygous for DP 1558NR B2RF and DP 1747NR B2XF in the PVP certificates. The type of resistance found with the PR-FM/ST cultivars resulted in less suppression of the *M*. *incognita* population than the other resistance groups. An explanation of this partial resistance may indicate heterogeneous plant populations with regard to just a single *M*. *incognita* resistant gene.

The HR and PR-PHY groups have the herbicide trait package of (glyphosate  +  glufosinate  +  2,4-D choline) tolerance, while the PR-FM/ST have the herbicide trait package of glyphosate (GT) or glyphosate  +  glufosinate (GL) tolerance. However, the dicamba tolerant herbicide trait (XF) is in great demand with cotton producers in the USA, and the only group with *M*. *incognita* resistance and dicamba tolerance is the NR-DP group and ST 5600 B2XF, which was not included in the analysis. In 2019, the dicamba-tolerant trait accounted for 72% of the planted cotton acreage in the USA, and only about 0.1% of these acres went to *M*. *incognita* resistant (NR-DP) cultivars (Agricultural Marketing Service – Cotton and Tobacco Program, 2019). PhytoGen brand cultivars with either partial or full *M*. *incognita* resistance accounted for 7%, and the PR-FM/ST group plus ST 5600 B2XF accounted for 2% of planted acres. The herbicide trait package is an important reason that cotton producers are not utilizing *M*. *incognita* resistant cultivars. Nonchemical control of *M*. *incognita* is possible using currently available commercial cultivars. However, the use of these nematode resistant cultivars is not currently widespread, in part because of differences in herbicide tolerant traits.
